# Pain Bloc-R Alleviates Unresolved, Non-Pathological Aches and Discomfort in Healthy Adults—A Randomized, Double-Blind, Placebo-Controlled, Crossover Study

**DOI:** 10.3390/nu12061831

**Published:** 2020-06-19

**Authors:** Malkanthi Evans, Abdul M. Sulley, David C. Crowley, Jamie Langston, Najla Guthrie

**Affiliations:** 1 KGK Science Inc., London, ON N6A 5R8, Canada; asulley@kgkscience.com (A.M.S.); dcrowley@kgkscience.com (D.C.C.); nguthrie@kgkscience.com (N.G.); 2 LifeSeasons, Inc., Springville, UT 84663, USA; Jamie@lifeseasons.com

**Keywords:** pain, ache, discomfort, natural health product, randomized controlled trial

## Abstract

The lack of effective treatment for chronic discomfort without negative side effects highlights the need for alternative treatments. Pain Bloc-R is a natural health product composed of vitamins B_6_, B_12_, D, white willow bark extract, Angelica root extract, acetyl L-carnitine HCl, caffeine, L-theanine, Benfotiamine, and L-tetrahydropalmatine. The objective of this study was to compare the effects of Pain Bloc-R, acetaminophen, and placebo on unresolved aches and discomfort as assessed by the brief pain inventory (BPI) and modified Cornell musculoskeletal discomfort questionnaires. This randomized, double-blind, placebo-controlled, crossover study consisted of three 7-day periods with Pain Bloc-R, acetaminophen, or placebo, each separated by a 7-day washout. Twenty-seven healthy adults (ages 22–63 years) were randomized to receive the three interventions in different sequences. The BPI “pain at its worst” scores were significantly lower when participants took Pain Bloc-R than when they took acetaminophen (21.8% vs. 9.8% decrease, *p* = 0.026) after seven days of supplementation. Pain Bloc-R achieved a significant improvement in the “pain at its least” score, significantly decreased the interference of discomfort in walking, and significantly decreased musculoskeletal discomfort total scores (34%, *p* = 0.040) after seven days. In a *post hoc* subgroup analysis based on age and gender, male participants ≤45 years taking Pain Bloc-R reported significant reductions in pain severity and pain interference vs. acetaminophen. Pain Bloc-R performed as well as acetaminophen in managing unresolved non-pathological pain in otherwise healthy individuals.

## 1. Introduction

Chronic pain is one of the most common reasons for adults to seek medical care [1]. Across geographies and ages, musculoskeletal conditions account for the greatest proportion of chronic pain [2]. It is estimated that half of the adults in the United States live with a musculoskeletal condition, thereby costing 213 billion dollars in 2011 [3]. Chronic, non-specific musculoskeletal pain is a leading cause of functional limitations [3], reducing quality of life and perceived health [3,4]. Therefore, it is important to develop efficient and safe pain management interventions.

Muscle pain and discomfort can result from strenuous activities. For example, muscle soreness can occur after days of unusual or particularly heavy work/exercise, which is characterized by stiffness, tenderness, and weakness of the musculature [5]. Another common example of unresolved discomfort is back muscle tension and pain induced by either anxiety or stress [6].

Common interventions to target and resolve pain include reducing anxiety and muscle tension, decreasing the inflammatory response, and reducing the sensation of pain by regulating hormonal responses and neurotransmitters. Acetaminophen is used weekly by nearly one quarter of Americans to manage non-specific and unresolved pain, making it the most commonly used pain prescription and over-the-counter drug in the United States [7]. It is considered a safe first-line therapy by the Federal Drug Administration when taken in recommended amounts [8]. However, its presence in a wide range of medications for diverse indications makes it easy for consumers to overdose, which can cause acute liver failure [9]. This ease of accidental overdose with acetaminophen is well-documented and has made it not only a potential cause of acute liver failure, but indeed the leading cause of acute liver failure in the United States [10]. Non-steroidal anti-inflammatory drugs (NSAIDs) are another standard management strategy to help alleviate unresolved pain or soreness and to restore function of muscles [11]. While NSAIDs have a lesser potential for hepatotoxicity than acetaminophen, current evidence discourages their use in patients with existing liver injury [12]. They may also elicit other unfavorable side effects, such as upper gastrointestinal tract symptoms, kidney injury, hypertension, and congestive heart failure [13]. These adverse events can be so severe that they lead to hospitalization, and have made NSAIDs one of the most common drug classes leading to preventable hospitalization, being responsible for 11% of preventable drug-related hospitalizations [14]. These side effects may become more prevalent or more severe with long term use [13], as they may be needed for a person with non-specific occupational pain. The lack of an effective intervention for chronic discomfort or soreness without negative side effects highlights an urgent need for an alternative treatment.

Pain Bloc-R is a natural health product composed of vitamin B_6_, vitamin B_12_, vitamin D, white willow bark extract, Angelica root extract, acetyl L-carnitine HCl, caffeine, L-theanine, Benfotiamine, and L-tetrahydropalmatine (L-THP). This cocktail of ingredients may ease discomfort by reducing inflammation and regulating hormones and neurotransmitters to promote muscle relaxation. Vitamin D, while not immediately considered for pain relief, has been shown to have anti-inflammatory effects in humans through regulation of cytokines, inhibition of nuclear factor-κB (NF-κB) and prostaglandins, and upregulation of mitogen-activated protein kinase 5 [15]. Similarly, white willow bark and *Angelica dahurica* root have been shown in animal models and in vitro to reduce inflammatory markers, including TNF-α, IL-6, and cyclooxygenase-2 (COX-2) [16,17]. L-theanine promotes the synthesis of the neurotransmitters gamma-aminobutyric acid (GABA) and dopamine, and plays a role in reducing anxiety and stress and one in promoting relaxation of muscle [18]. L-THP, a novel ingredient isolated from Chinese herbal medicine, has demonstrated an anti-hyperalgesic effect in mice, potentially by enhancing the function of dopamine receptors [19,20]. Caffeine has been found to improve pain relief when used as an adjuvant to conventional analgesics [21]. The acetyl L-carnitine HCl and Benfotiamine in Pain Bloc-R also have shown antioxidant effects in rats, which may reduce inflammatory responses [22,23].

This randomized, double-blind, placebo-controlled, crossover study evaluated the efficacy of Pain Bloc-R in the management of unresolved, non-pathological aches and discomfort in otherwise healthy adults, which was assessed by brief pain inventory (BPI) and modified Cornell musculoskeletal discomfort (MD) questionnaires. In addition, product perception and tolerability were assessed by a product perception questionnaire. The effects of Pain Bloc-R were compared with acetaminophen as a comparator and placebo.

## 2. Materials and Methods

### 2.1. Study Design

This study was approved by the Natural and Non-Prescription Health Products Directorate, Health Canada, Ottawa, Ontario on 25 April 2019. Research ethics board approval was granted on 10 May 2019 from the Institutional Review Board (IRB) Services, Aurora, Ontario. The study was conducted in compliance with the International Council for Harmonization of Technical Requirements for Pharmaceuticals for Human Use (ICH) Guideline for Good Clinical Practice (GCP) and in accordance with the Declaration of Helsinki guidelines and its subsequent amendments. The trial was registered at Clinicaltrials.gov (NCT03965819) and followed the CONSORT guidelines for randomized controlled trials [24] (Supplementary Table S1).

This randomized, double-blind, placebo-controlled, crossover study was carried out at KGK Science Inc. clinic site, London, ON, Canada from 23 May 2019 to 29 August 2019. All participants in the study provided written informed consent at the screening visit, prior to any study procedures being initiated.

Participants met the following inclusion criteria: male or female; aged 18–65 years; BMI 18.0–32.5 kg/m^2^; experiencing unresolved aches and discomfort for at least two weeks due to occupational (e.g., manual laborer) or non-occupational activities; and willing to undergo a washout prior to enrollment if taking non-prescription medication. All participants agreed to complete questionnaires and diaries associated with the study and to comply with study procedures.

Individuals were excluded if they suffered from a chronic disease condition causing chronic pain; used prescribed medications to relieve the pain and discomfort; had uncontrolled blood pressure or sugar levels; were cognitively impaired and/or unable to give informed consent; or had any other condition which in the medical investigator’s opinion may have adversely affected the individual’s ability to complete the study or its measures, or which may have posed significant risk to the individual.

Clinical and qualitative assessments were conducted at all study visits. At all visits, participants’ diaries were reviewed for concomitant therapies, adverse events, and product use. The BPI and Cornell MD questionnaires were administered at the start and end of each 7-day study period, and a product perception questionnaire was administered at the end of each study period. Study diaries, the investigational products, and rescue medication were dispensed on the first day of each study period. Vital signs and anthropometric measurements were taken at each visit while laboratory parameters for all safety endpoints were assessed at the screening and end of each 7-day period.

### 2.2. Investigational Product

Pain Bloc-R consisted of the ingredients listed in Table 1. The comparator acetaminophen (DIN 02447479) contained non-medical ingredients similar to those present in the investigational product (rice bran, hypromellose, titanium dioxide, and sodium copper chlorophyllin, carnauba wax, polyethylene glycol, povidone, pregelatinized starch, and stearic acid) and was encapsulated for blinding purposes (325 mg/capsule). The placebo was composed entirely of non-medical ingredients (rice bran, hypromellose, titanium dioxide, and sodium copper chlorophyllin). Pain Bloc-R, acetaminophen, and placebo were presented in non-gastro-resistant, green veggie capsules (hypromellose, titanium dioxide, and sodium copper chlorophyllin), without differences in size, color, taste, texture, or packaging.

**Figure 1:**
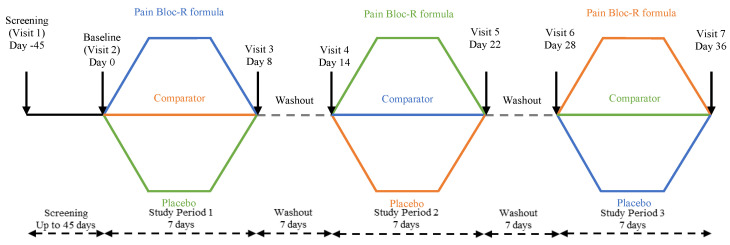
Study design. The study design was a randomized, double blind, placebo-controlled, crossover study. Healthy participants (*n* = 27) were randomized equally to each of the study arms. The trial consisted of three intervention periods and two wash-out periods, lasting a total of five continuous weeks.

The study consisted of three 7-day supplementation periods with Pain Bloc-R, acetaminophen, and placebo respectively, each separated by a 7-day washout period (Figure 1).

All participants who met all inclusion without meeting any exclusion criteria at screening and baseline were randomized into study groups. During the supplementation period, one group received Pain Bloc-R, a second group received acetaminophen, and a third group received placebo. Each participant was dispensed their capsules in two bottles. The bottles that were dispensed to the Pain Bloc-R and placebo groups contained the investigational product and placebo, respectively. For the comparator group, one bottle contained acetaminophen (325 mg) and the other bottle contained the placebo. Two bottles were used to maintain blinding and allocation concealment, whereby all participants were consistently required to take two capsules during each period of the study. A dose of 325 mg acetaminophen was selected because the rescue medication provided was also 325 mg of acetaminophen. By only providing 325 mg as the comparator dose, participants could take rescue medication as needed with minimal concern of overdose. In all cases, participants were instructed to take one capsule from each bottle at the time of day when discomfort is most often felt, with or without meals, beginning on the day following randomization (day 1). Participants were instructed not to take more than one capsule from each bottle per day. If participants were experiencing pain two hours after taking their daily maximal dose of one capsule from each bottle, they were advised to take the rescue medication (regular strength acetaminophen capsules) without exceeding nine capsules per day. Participants were asked not to double-up on any unused capsules from a previous day.

### 2.3. Randomization and Blinding

Eligible participants were assigned a randomization number by a blinded investigator per the order of the randomization list (www.randomization.com). Each randomization code represented an allocation to a dosing arm of the study. Following randomization participants were identified by their initials and date of birth and were assigned a participant number at their screening visit.

The placebo and comparator product were matched to the investigational product and contained similar excipient to ensure blinding. The investigational product, comparator, and placebo were sealed in bottles that were identical in appearance and labelled per the requirements of ICH GCP guidelines and applicable local regulatory guidelines. Unblinded personnel not involved in any study assessments labelled the investigational product. A randomization schedule was created and provided to the investigator indicating the order of randomization. Investigators, other site personnel, and participants were blinded to the products.

### 2.4. Compliance

Participants were instructed to save all packages (unused or open) and return them at each visit to the clinic for the determination of compliance. Product compliance was determined by the number of consumed dosage units divided by the number of dosage units expected to have been taken, multiplied by 100. In the event of a discrepancy between the information in the study diary and the amount of study product returned, compliance was based on the product returned unless an explanation for the loss of product was provided. Participants found to have a compliance of <80% or >120% were counselled while those outside this range were considered as non-compliant.

### 2.5. Outcomes

The study compared the effects of Pain Bloc-R, acetaminophen, and placebo on unresolved aches and discomfort after 7 days of supplementation.

### 2.6. Brief Pain Inventory (BPI) Questionnaire

The BPI questionnaire, originally developed by Cleeland and Ryan in 1994 and now distributed by MD Anderson Cancer Centre, is a 9-item self-administered assessment tool used to score an individual’s level of discomfort, discomfort severity and discomfort interference of unresolved aches and discomfort [25]. Part A of the questionnaire evaluates pain severity and asks the participants to rate their “worst,” “least,” “average,” and “right now” levels of pain on a scale of 0 (no pain) to 10 (as bad as you can imagine). Part B evaluates the pain interference levels and asks the participants to rate the impact of their pain on their general activity, mood, walking ability, normal work, relationships with other people, sleep, and enjoyment of life. Together, parts A and B aim to quantify pain levels and the resulting quality of life. The questionnaire was administered at each visit to monitor changes in participant’s pain over the course of each period.

### 2.7. Modified Cornell Musculoskeletal Discomfort Questionnaire

The Modified Cornell MD questionnaire, developed by the Human Factors and Ergonomics Laboratory at Cornell University, is used to assess physical discomfort and its impact on quality of life [26]. The questionnaire includes a list and image of body parts where discomfort could be experienced. This study used a slightly modified version of the questionnaire to exclude the word “pain,” as the study focused on participant discomfort felt by an otherwise healthy population. The questionnaire assessed the discomfort frequency scores of 0 (never), 1.5, 3.5, 5, and 10 (several times a day). The questionnaire then assessed discomfort severity score: whether the aches and discomfort were “slightly,” “moderately,” or “very” uncomfortable. Finally, the questionnaire determined whether the discomfort interfered with the participants’ day-to-day lives: “not at all,” “slightly interfered,” or “interfered a lot.” The discomfort score was calculated by multiplying the individual frequency, severity, and interference scores for each body part, and the products for the body parts for each participant were summed to yield a total discomfort score.

### 2.8. Laboratory Analyses

Safety endpoints were analyzed from the blood drawn at screening, and at day 8, day 22, and day 36 by Dynacare (London, Ontario, Canada) using standardized procedures. The tests included the analysis of hemoglobin, hematocrit, platelet count, red blood cell count (RBC), red blood cell indices, red cell distribution width (RDW), white blood cell count (WBC), differentials (neutrophils, lymphocytes, monocytes, eosinophils, basophils), a liver function test (AST, ALT, total bilirubin), and a kidney function test (creatinine). Urine pregnancy tests were conducted at the KGK clinic for participants of childbearing potential at screening and baseline (day 0).

### 2.9. Adverse Events

During the study, participants recorded any adverse event (AE) in their diary. AEs were documented in the study record and were classified as per the description, duration, intensity, frequency, and outcome. The medical investigator determined causality and intensity of all reported AEs (if appropriate). AEs were coded with the Medical Dictionary for Regulatory Activities terminology (MEDRA) System Organ Class, version 22.0.

### 2.10. Statistical Analysis

Being a pilot study, and following the rule of thumb of having at least 12 participants for a pilot study [27], a sample size of 27 participants was enrolled in the study, accounting for 20% attrition rate. *Post hoc* power calculations based on the per protocol population indicated that the minimally detectable differences in mean change in BPI “pain at its worst” score and BPI pain severity scores between any two groups were 1.61 and 0.89 respectively, given pooled standard deviations of 1.72 and 0.95 respectively, and given 80% power and 5% alpha. A sample size of 27 participants was enrolled in the study accounting for 20% attrition rate.

The per protocol (PP) population consists of all participants who consumed at least 80% of intervention doses, did not have any major protocol violations, and completed all study visits and procedures connected with measurement of the primary variable. Only observed values were used for the analysis of the PP population. No imputation was performed for the PP population or for safety outcomes. Variables were tested for normality and log-normality, where log-normality distributed variables were analyzed in the logarithmic domain. Appropriate non-parametric tests were used to analyze intractably non-normal variables.

The primary outcome was the change in unresolved aches and discomfort from pre-supplementation to day 7 between Pain Bloc-R, acetaminophen, and placebo as assessed by the scores derived from the BPI questionnaire. Secondary outcomes included (1) the changes in musculoskeletal discomfort (modified Cornell MD questionnaire) and (2) general discomfort (BPI questionnaire) scores from pre-supplementation to day 7 post-supplementation between Pain Bloc-R, acetaminophen, and placebo; (3) product perception; and (4) proportion of participants who consumed rescue medicine. Assessment of the scores for the primary and secondary outcomes 1–3 was conducted using mixed models. The models included intervention, sequence, and period as fixed effects, and subject as a random effect. Between-group *p* values were obtained from the model. Within-group *p* values for differences between pre and post-supplementation were obtained using paired Student’s *t* tests or Wilcoxon signed rank tests as appropriate. For secondary outcome 4, the proportion of participants consuming rescue medicine during the trial, was analyzed using a generalized estimating equations model. The model assumed binary distribution and included intervention, sequence, and period as fixed effects. A *post hoc* analysis on the effects of age and age by gender were explored by examining the respective subgroups. The subgroup analyses were conducted using the same methods described above.

All statistical analyses were completed using R version 3.5.3 [28], RStudio version 1.2.1335 [29] for Microsoft Windows, nlme package (for mixed models) [30] and related packages. Probabilities ≤0.05 were considered statistically significant.

## 3. Results

### 3.1. Study Participant Dispositions

Participants ranged in age from 22–63 years, were mostly females, and were predominantly Western European White (Table 2). All participants were deemed healthy based on the assessment of their anthropometric parameters, vital signs (Table 2), and metabolic panel measurements (Table 3). Of the 42 participants who were screened, twenty-seven eligible participants were randomized to receive three interventions in one of three sequences, with nine participants per sequence. Participants were similar in their demographics between the three sequences (except *p* < 0.05 for weight and body mass index (BMI), data not shown).

**Figure 2:**
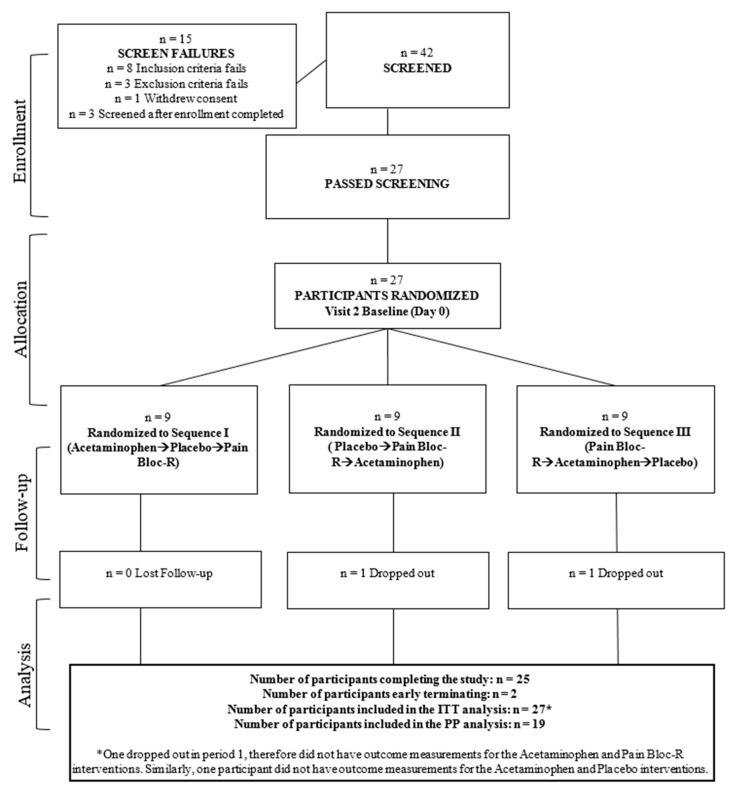
Disposition of study participants.

A total of 41 protocol deviations occurred in this study and eight participants were excluded from the PP analysis. Six participants who did not take rescue medication as directed and two participants who withdrew from the study were excluded from the PP population. The disposition of participants through the study is shown in Figure 2. The mean compliance for the three sequences was >95%, and not significantly different between products.

### 3.2. General Pain Severity

**Figure 3:**
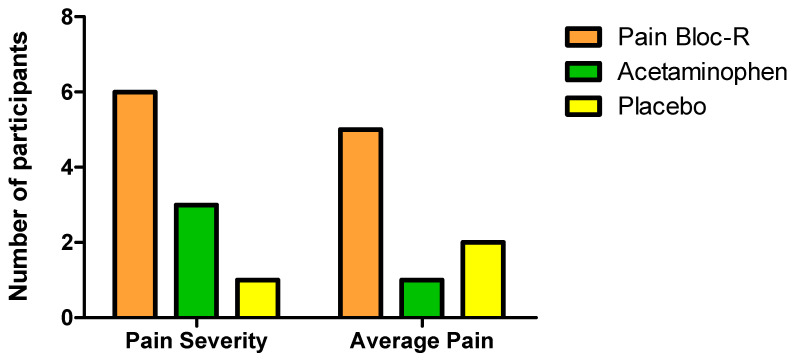
Number of participants reaching a minimal clinically important improvement based on the BPI pain severity scores (at least 32.3%) and average pain scores (at least 27.7%) in the per protocol (PP) population.

Based on the pain severity subscale scores assessed by the BPI, 32% of participants taking Pain Bloc-R, 16% taking acetaminophen, and 5% of those taking placebo achieved the minimal clinical important improvement (MCII) of a 32.3% reduction in overall pain severity [31] (Figure 3). MCII is defined as the smallest improvement in an outcome that is deemed to be important to a patient or participant population [32]. Participants on Pain Bloc-R or acetaminophen had 26% and 11% greater improvements in their overall pain severity scores, respectively, compared to placebo.

A total of 26% of participants taking Pain Bloc-R, 5% of those taking acetaminophen, and 11% of those taking placebo achieved the MCII of 27.7% required to show clinical relevance in response to the interventions based on average pain subscale scores (Figure 3). Participants on Pain Bloc-R experienced 16% greater relief of their average pain than those on placebo while those on acetaminophen experienced 5.2% less relief than those taking placebo.

There were no significant differences in the change of scores for pain severity after seven days between interventions. There were significant decreases in pain severity scores with Pain Bloc-R and acetaminophen (*p* = 0.024 and *p* = 0.010, respectively) from baseline to day 7 (Table 4).

A *post hoc* analysis of a subgroup based on age revealed a significant 25.9% decrease from baseline in average pain score for participants ≤45 years old taking Pain Bloc-R (*p* = 0.046). These participants also reported a significant decrease in pain severity after taking Pain Bloc-R or acetaminophen for seven days (*p* < 0.05). There were no significant changes in general pain severity reported by participants >45 years of age.

**Figure 4:**
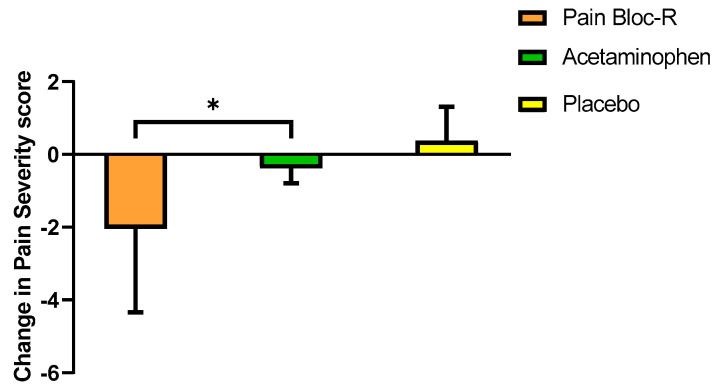
Change in pain severity score from pre-supplementation to post-supplementation (day 7) in the ≤45 years of age male PP population (*n* = 6). n, number; SD, standard deviation. Between group *p*-values were generated using a linear mixed effect model with treatment, sequence, and period as fixed effects and subject as a random effect. Between group *p*-values were adjusted using Tukey’s HSD. * *p* ≤ 0.05 considered statistically significant.

When participants ≤45 years of age were further sub-grouped by gender, males reported a significant decrease in pain severity score when taking Pain Bloc-R compared to acetaminophen (*p* = 0.026) (Figure 4).

### 3.3. Interference with Daily Functioning

There were no significant differences in the changes of scores for pain interference from baseline to day 7 between interventions, with the exception of less pain interference in walking ability with Pain Bloc-R than with acetaminophen (*p* = 0.005). The decrease of pain interference in walking ability with Pain Bloc-R was also trending towards significance (*p* = 0.068), which translated into a significant 36.7% improvement compared to acetaminophen (*p* = 0.005). A significant improvement from baseline to day 7 in the enjoyment of life score was only reported with acetaminophen (*p* = 0.020). Participants taking acetaminophen (*p* = 0.032) and placebo (*p* = 0.022) reported significant relief due to pain treatments. For participants ≤45 years, acetaminophen also resulted in significant relief due to pain treatment after seven days (*p* = 0.036).

There was a significant decrease in pain interference score for participants ≤45 years old taking Pain Bloc-R (*p* = 0.045) from baseline to Day 7. In these participants, Pain Bloc-R significantly decreased pain interference with enjoyment of life compared to acetaminophen (*p* = 0.019). Participants taking acetaminophen or placebo had decreases in pain interference with relationships with other people (8.3% and 39.2%, respectively), which resulted in significant differences between acetaminophen vs. Pain Bloc-R (*p* = 0.010) and placebo vs. acetaminophen (*p* = 0.003).

**Figure 5:**
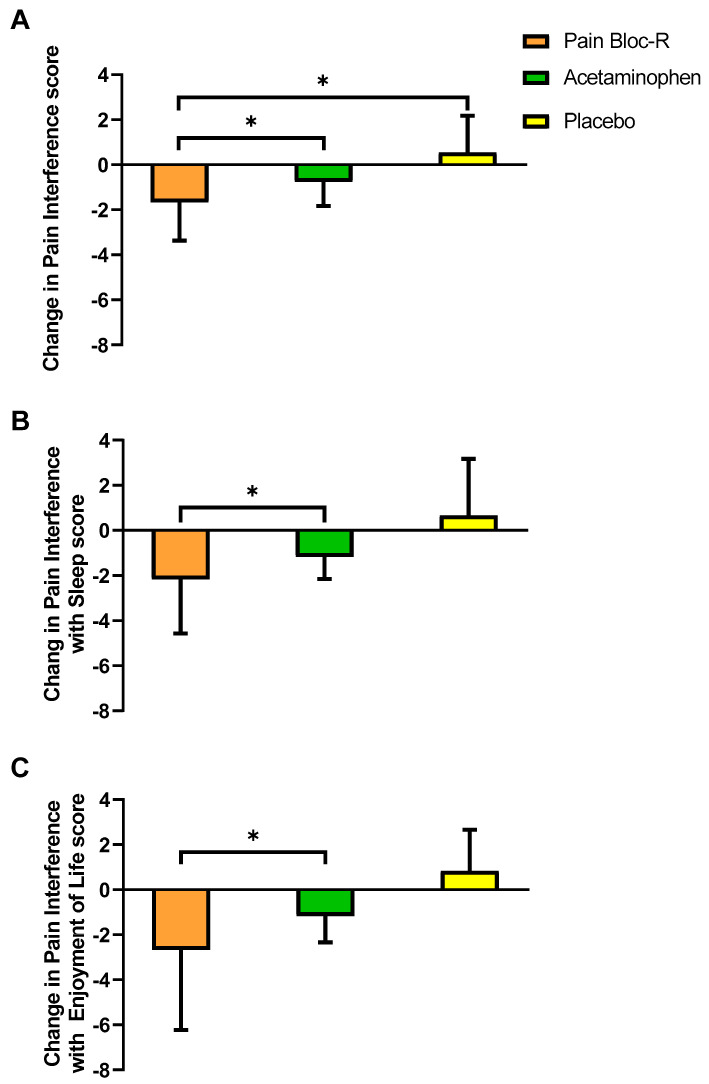
Change in (**A**) pain interference score, (**B**) pain interference with sleep score, (**C**) pain interference with enjoyment of life score from pre-supplementation to post-supplementation (day 7) in the ≤45 years of age male PP population (*n* = 6). n, number; SD, standard deviation. Between group *p*-values were generated using a linear mixed effect model with treatment, sequence, and period as fixed effects and subject as a random effect. Between group *p*-values were adjusted using Tukey’s HSD. * *p* ≤ 0.05 considered statistically significant.

Male participants ≤45 years of age taking Pain Bloc-R reported a significant decrease in pain interference vs. acetaminophen (*p* = 0.036) or placebo (*p* = 0.008) (Figure 5A). Moreover, pain interference with sleep (*p* = 0.011, Figure 5B) and enjoyment of life (*p* = 0.012, Figure 5C) was decreased with Pain Bloc-R vs. acetaminophen in these participants. Consistent with the observation for male participants, females ≤45 years of age taking Pain Bloc-R reported significantly improved pain interference with sleep vs. acetaminophen (*p* = 0.007).

In male participants >45 years of age, pain interference with enjoyment of life was decreased with Pain Bloc-R (*p* = 0.016) and placebo (*p* = 0.037). In these participants, placebo significantly decreased pain interference (*p* = 0.008) and interference with general activity (*p* = 0.035) after seven days. Acetaminophen significantly decreased pain interference with mood (*p* = 0.045) from baseline to day 7. Placebo significantly decreased pain interference with mood vs. Pain Bloc-R in female participants >45 years of age (*p* = 0.015).

### 3.4. Management of Unresolved Aches and Discomfort

In the PP population, “pain at its worst” scores were significantly lower when participants were taking Pain Bloc-R compared to acetaminophen (21.8% vs. 9.8% decrease, *p* = 0.026). There were significant decreases in “pain at its worst” with Pain Bloc-R (*p* = 0.016) and acetaminophen (*p* = 0.037), respectively, after seven days of supplementation (Table 5). With Pain Bloc-R, there was also a significant decrease in “pain at its least” (29.2%, *p* = 0.035) and a trend for a decrease in “pain you have right now” (24.8%, *p* = 0.052). In participants ≤45 years of age, there were significant decreases in “pain at its least” score with Pain Bloc-R (48.7%, *p* = 0.046) and acetaminophen (37.9%, *p* = 0.049) from baseline to day 7.

### 3.5. Musculoskeletal Discomfort

Using the Modified Cornell MD questionnaire, it was reported that the most common body areas with aches or discomfort were the neck (52.6% of participants), right shoulder (42.1%), upper back (47.4%), lower back (63.2%), and hip/buttocks (52.6%). No significant differences between interventions were reported for changes in muscular discomfort total scores or scores for specific sites, from baseline to day 7. However, musculoskeletal discomfort total body score only decreased significantly with Pain Bloc-R (34%, *p* = 0.040) after seven days (Table 6). Treatment with Pain Bloc-R lowered discomfort at each of the most common pain sites listed except for the upper back, although the differences were not significant. In contrast, participants on acetaminophen reported a non-significant decrease in neck (21%) and right shoulder (6.5%) discomfort but a significant increase in lower back discomfort (46%, *p* = 0.024). There were no differences in MD score based on age or age by gender subgroups, with the exception of a decrease in total MD total score with acetaminophen from baseline to day 7 for participants ≤45 years of age (*p* = 0.028).

### 3.6. Use of Rescue Medicine and Product Perception

No significant differences were reported for the proportion of participants consuming rescue medication or product perception and tolerability at day 7 between interventions.

### 3.7. Safety Outcomes

There were no clinically relevant changes in the vital signs, hematology, kidney or liver markers, or electrolytes from baseline values (Table 2 and Table 3).

Thirty-six AE were reported by 15 participants in this study. Of these 24 were reported by participants on Pain Bloc-R, nine by those taking acetaminophen, and nine while on placebo. Twenty of these events were classified as “not related” or “unlikely” related while 16 were classified as “possibly” related to the interventional products. Of the latter, 10 (dizziness *n* = 2, nausea *n* = 3, drowsiness *n* = 4, and stomach pain *n* = 1) were reported while participants were on Pain Bloc-R, five (upset stomach *n* = 1, drowsiness *n* = 2, diarrhea *n* = 1, dizziness *n* = 1) while they were on acetaminophen, and one while on placebo. All AE were resolved by the end of study, except for one abnormal liver function test, which was deemed to be “unlikely” to be related to the investigational product and was resolved 28 days after the study.

## 4. Discussion

This randomized, double-blind, placebo-controlled, crossover study evaluated the efficacy of Pain Bloc-R in reducing unresolved, non-pathological aches and discomfort. Pain Bloc-R performed and acetaminophen in managing unresolved aches and discomfort, and even better than acetaminophen in reducing the pain interference in walking ability. Seven days of Pain Bloc-R supplementation led to significant reductions of 21.8% in pain severity and 34.1% in musculoskeletal discomfort without serious adverse events or significant changes in safety parameters. Furthermore, more participants reported clinically meaningful reductions in average pain and pain severity with Pain Bloc-R as compared to acetaminophen or placebo, with six participants reporting the MCII when taking Pain Bloc-R, three when taking acetaminophen, and only one when taking placebo. These findings support the potential role of Pain Bloc-R in managing unresolved aches and discomfort, as a safe and effective alternative to acetaminophen.

Although both Pain Bloc-R and acetaminophen led to reductions in pain severity and interferences with certain daily functioning from pre to post-supplementation, the improvement in “pain at its worst” was significantly greater with Pain Bloc-R than acetaminophen (21.8% vs. 9.8%). This indicates the ability of Pain Bloc-R to reduce the magnitude of the aches and discomfort experienced by the participants, and this may have contributed to the significant 36.7% improvement in their walking score compared to when taking acetaminophen. It is also notable that with Pain Bloc-R, more participants reached an improvement beyond the MCII threshold, defined as the smallest improvement in the outcome that is deemed meaningful by participants [32], for BPI pain severity scores and average pain scores than acetaminophen.

Discomfort in neck, right shoulder, upper back, lower back, and hip/buttocks was all prevalent in the study population. This is consistent with current evidence that shoulder and back pain are the common types of pain in the adult population [33]. With Pain Bloc-R, the 34% decrease in total musculoskeletal discomfort was supported by the lower discomforts in almost all the studied body parts except upper back. In comparison, acetaminophen did not achieve similar pain-alleviating effects.

Interestingly, when subgroups based on age were examined in a *post hoc* analysis, participants ≤45 years of age reported significant reductions in pain interference with enjoyment of life when taking Pain Bloc-R compared to acetaminophen. Pain response including pain severity, average pain and “pain at its least” were significantly reduced for those participants after taking Pain Bloc-R for seven days. Acetaminophen also resulted in significant reductions in pain severity and “pain at its least” after seven days for participants ≤45 years of age. The effect of age on pain perception is inconsistent in existing literature and varies depending on the stimuli [34]. Further, there was a differential response to Pain Bloc-R based on gender within each age group. Studies have reported that women more frequently report pain than men [35], and the perception that women have a greater sensitivity to pain and more willingness to report pain [36]. Surprisingly, in the current study it was the male participants ≤45 years of age that reported significant improvements in overall pain interference, pain interference with sleep and enjoyment of life with Pain Bloc-R compared to acetaminophen. Previous studies have demonstrated gender-based differences in response to vitamin D, a Pain Bloc-R ingredient, in immune function [37] and cardiometabolic biomarkers [38]. The potential relationship between pain relief and age/gender observed in the current study warrants further investigation with a more robust sample size.

The ability of Pain Bloc-R to deliver similar or better pain relief than acetaminophen is an important finding of this study. Acetaminophen is well established as a pain-relieving drug that exerts its analgesic effect by inhibiting cyclooxygenase (COX), which is responsible for the production of prostanoids [39,40]. Therefore, acetaminophen does not reduce inflammation or remedy the cause of pain, but only masks the sensation [40]. Currently, there have been no studies investigating the pain-alleviating effect of a formula with similar composition of Pain Bloc-R. However, with a variety of analgesic ingredients, Pain Bloc-R matched the efficacy of acetaminophen potentially by targeting multiple pain pathways with its ingredients. Vitamin D [15], white willow bark [16], and Angelica root extract [17] serve as anti-inflammatory agents. The antioxidant properties of acetyl L-carnitine and Benfotiamine can potentially reduce neuroinflammation and alleviate pain caused by peripheral neuropathy [41]. L-theanine can increase the release of dopamine [42], while L-tetrahydropalmatine (L-THP) can block dopamine uptake [19,20], thereby modulating pain perception.

A novel ingredient in Pain Bloc-R is L-THP, which is an active constituent extracted from plant species of the genera *Stephania* and *Corydalis* [20]. Gaining recent popularity for its demonstrated potential in the treatment of cocaine addiction, L-THP has been shown to be a potent antagonist of dopamine receptors D1 and D2, and displays activity at other dopamine and serotonin receptors [20]. The analgesic and antinociceptive effects of L-THP have been demonstrated in several preclinical rodent studies [43,44,45]. The safety of L-THP has been examined and resulted in minimal changes of liver enzyme activities in dogs [46] and similar numbers of side effects compared with placebo in a 3.5-day intervention with 20 cocaine users [47]. Overall, Pain Bloc-R was safe and well-tolerated in the present study as determined by the low incidence of adverse events, all but one of which were resolved by the end of the study, and all hematology and clinical chemistry profiles remained within clinically normal ranges. In addition to these objective measures of product safety, participants felt that it had either a neutral or beneficial impact on their health, and felt they tolerated it well, as assessed by a favorable rating of 19.74 out of 35 on the product perception questionnaire. This rating was not significantly different between any groups, indicating that participants felt that Pain Bloc-R achieved its purpose as well as acetaminophen, and was similarly tolerable.

The population in this study consisted of 27 healthy adults aged 22–63 years presenting with non-specific pain that had persisted for at least two weeks prior to baseline. It was not required that pain be linked to employment, but construction workers, manual laborers, and athletes were targeted for recruitment. This population was selected because these people are more likely to experience persistent non-specific pain due to repetitive exertions that workers are required to perform [48]. This population is also an important one to study because they make up a large portion of the workforce, while unresolved aches and discomfort are a main cause of functional limitations and lead to economical and medical burdens. As of October 2019, about 21 of 155 million Americans in the labor force were employed in the construction, manufacturing, and natural resources and mining sectors [49]. Developing a safe and effective management intervention for aches and discomfort is critical for this population.

The strengths of the present study include the rigorous double-blind, randomized, crossover design, the extensive evaluation of aches and discomfort and assessment of safety, and the conservative statistical approach. The use of acetaminophen as a comparator also provided further insights into the advantages of Pain Bloc-R. The limitations of this study include a small sample size and little variation in participant demographics. For future studies, Pain Bloc-R should be investigated in a larger and more diverse population, with a sample size calculated based on the results of the current study. In addition, given that NSAIDs are another common option for the management of chronic aches and discomfort, futures studies should include other comparators such as ibuprofen and other anti-inflammatory medications.

## 5. Conclusions

In conclusion, aches and discomfort were decreased compared to pre-supplementation with Pain Bloc-R, and participants reported lowered interference with walking ability due to pain. Clinically meaningful reductions in average pain and pain severity were experienced in participants while on Pain Bloc-R as compared to acetaminophen or placebo. Pain Bloc-R was found to be safe and efficacious and should be considered as a viable alternative for the relief of unresolved, non-pathological aches and discomfort.

## Figures and Tables

**Table 1:** Composition of Pain Bloc-R.

Ingredient	Quantity (per Capsule) *
Vitamin D_3_ (as cholecalciferol)	500 IU
Vitamin B_12_	0.5 mg
White willow bark extract (std.to 15% salicin) (Salix alba)	150 mg
Angelica root (Angelica dahurica)	50 mg
Acetyl L-carnitine HCl	50 mg
Caffeine (from Green Coffee bean, Coffea arabica)	37.5 mg
L-Theanine	37.5 mg
BenfoPure Benfotiamine	25 mg
Pyridoxal 5 Phosphate	17 mg
L-Tetrahydropalmatine	25 mg

Non-medical ingredients: rice bran, hypromellose, titanium dioxide, sodium copper chlorophyllin, maltodextrin, modified food starch (corn), sucrose, and silicon dioxide. * Participants were instructed to take two capsules of Pain Bloc-R per day.

**Table 2:** Baseline characteristics for all participants enrolled (*n* = 27).

Demographic Characteristics	
Characteristics	Mean ± SD
Age (years)	41.44 ± 11.60
Gender (n)	
Female	15
Male	12
Systolic Blood Pressure (mmHg)	121.96 ± 11.96
Diastolic Blood Pressure (mmHg)	76.15 ± 8.41
Heart Rate (bpm)	67.46 ± 11.43
Weight (kg)	78.33 ± 16.33
BMI (kg/m^2^)	26.22 ± 3.60
Ethnicity [n(%)]	
Eastern European White	1 (3.70%)
Hispanic or Latino	1 (3.70%)
South American	1 (3.70%)
Western European White	24 (88.90%)

n, number; SD, standard deviation.

**Table 3:** Hematology and clinical chemistry markers for all participants enrolled (*n* = 27).

Screening Hematology and Clinical Chemistry	
Parameter	Mean ± SD
Alanine Aminotransferase (ALT) (U/L)	21.56 ± 9.81
Aspartate Aminotransferase (AST) (U/L)	21.11 ± 7.20
Sodium (mmol/L)	140.78 ± 2.08
Potassium (mmol/L)	4.68 ± 0.51
Chloride (mmol/L)	103.07 ± 1.69
Estimated Glomerular Filtration Rate (eGFR) (mL/min/1.73)	99.19 ± 15.54
Creatinine (µmol/L)	73.00 ± 14.25
Bilirubin (µmol/L)	8.60 ± 4.63
Random Glucose (mmol/L)	5.03 ± 0.49
TSH (mIU/L)	1.84 ± 0.85
Glycated Hemoglobin (HbA1c) (%)	5.26 ± 0.30
White Blood Cell count (×10^9^/L)	5.51 ± 1.27
Platelets Count (×10^9^/L)	236.70 ± 51.05
Red Blood Cell count (×10^12^/L)	4.69 ± 0.44
Hemoglobin (g/L)	143.67 ± 11.77

n, number; SD, standard deviation; TSH, thyroid stimulating hormone.

**Table 4:** The BPI pain severity score and change in pain severity score from pre-supplementation to day 7 in the PP population (*n* = 19).

	Pain Bloc-R Mean ± SD Median (Min − Max)	Acetaminophen Mean ± SD Median (Min − Max)	Placebo Mean ± SD Median (Min − Max)
Pre-supplementation	4.76 ± 1.84	4.47 ± 1.47	4.09 ± 1.84
	4.75 (0.00 to 7.75)	4.75 (1.25 to 6.50)	4.50 (0.00 to 7.00)
Post-supplementation (day 7)	3.72 ± 1.89	3.97 ± 1.59	4.07 ± 1.96
	3.50 (0.50 to 6.25)	4.25 (0.75 to 7.00)	4.00 (0.50 to 7.25)
Change from Pre-supplementation to Post-supplementation (day 7)	−1.04 ± 1.84	−0.50 ± 0.75	−0.03 ± 1.11
	−1.00 (−6.00 to 1.00)	−0.50 (−2.25 to 0.75)	0.00 (−3.25 to 1.75)
Within Group *p*-Value ^+^	0.024	0.010	0.919
Between Group *p*-Value *	0.103 (r)	0.288 (r)	0.548 (r)

n, number; SD, standard deviation; Min, minimum; Max, maximum. ^+^ Within group *p*-values generated by the paired *t*-test. * Between group *p*-values were generated using a linear mixed effect model with treatment, sequence, and period as fixed effects and subject as a random effect. Between group *p*-values were adjusted using Tukey’s HSD. Between group *p*-values are acetaminophen versus Pain Bloc-R in column A, acetaminophen versus placebo in column B, and placebo versus Pain-Bloc-R in column C. (r) indicates values were ranked prior to generating ANOVA or ANCOVA.

**Table 5:** The BPI “pain at its worst” score and change in “pain at its worst” score from pre-supplementation to day 7 in the PP population (*n* = 19).

	Pain Bloc-R Mean ± SD Median (Min - Max)	Acetaminophen Mean ± SD Median (Min - Max)	Placebo Mean ± SD Median (Min - Max)
Pre-supplementation	6.05 ± 2.15	5.89 ± 1.66	5.53 ± 2.41
	6.00 (0.00 to 9.00)	6.00 (3.00 to 9.00)	6.00 (0.00 to 9.00)
Post-supplementation (day 7)	4.74 ± 2.26	5.32 ± 1.97	5.37 ± 2.67
	5.00 (0.00 to 8.00)	5.00 (1.00 to 8.00)	6.00 (0.00 to 9.00)
Change from Pre-supplementation to Post-supplementation (day 7)	−1.32 ± 2.16	−0.58 ± 1.12	−0.16 ± 2.17
	−1.00 (−8.00 to 2.00)	−1.00 (−2.00 to 1.00)	0.00 (−8.00 to 3.00)
Within Group *p*-Value ^+^	0.016	0.037	0.754
Between Group *p*-Value *	0.026 (r)	0.185 (r)	0.345 (r)

n, number; SD, standard deviation; Min, minimum; Max, maximum. + Within group *p*-values generated by the paired *t*-test. * Between group p-values were generated using a linear mixed effect model with treatment, sequence, and period as fixed effects and subject as a random effect. Between group *p*-values were adjusted using Tukey’s HSD. Between group *p*-values are acetaminophen versus Pain Bloc-R in column A, acetaminophen versus placebo in column B, and placebo versus Pain-Bloc-R in column C. (r) indicates values were ranked prior to generating ANOVA or ANCOVA.

**Table 6:** The modified Cornell musculoskeletal discomfort (MD) total score and change in MD score from pre-supplementation to day 7 in the PP population (*n* = 19).

	Pain Bloc-R Mean ± SD Median (Min - Max)	Acetaminophen Mean ± SD Median (Min - Max)	Placebo Mean ± SD Median (Min - Max)
Pre-supplementation	125.50 ± 105.85	121.03 ± 93.01	117.28 ± 105.23
	81.50 (6.00 to 313.00)	121.00 (14.50 to 363.50)	59.25 (10.00 to 358.00)
Post-supplementation (day 7)	82.76 ± 87.75	100.97 ± 76.37	103.61 ± 93.37
	43.00 (1.50 to 291.50)	72.00 (10.00 to 249.00)	67.50 (1.50 to 270.50)
Change from Pre-supplementation to Post-supplementation (day 7)	−42.74 ± 78.86	−20.05 ± 51.67	−8.00 ± 41.36
	−19.00 (−291.50 to 21.50)	−17.00 (−147.50 to 96.00)	−1.50 (−104.50 to 69.50)
Within Group *p*-Value ^+^	0.040	0.058	0.372
Between Group *p*-Value *	0.345 (r)	0.712 (r)	0.563 (r)

n, number; SD, standard deviation; Min, minimum; Max, maximum. ^+^ Within group *p*-values generated by the paired *t*-test. * Between group *p*-values were generated using a linear mixed effect model with treatment, sequence, and period as fixed effects and subject as a random effect. Between group *p*-values were adjusted using Tukey’s HSD. Between group *p*-values are acetaminophen versus Pain Bloc-R in column A, acetaminophen versus placebo in column B, and placebo versus Pain-Bloc-R in column C. (r) indicates values were ranked prior to generating ANOVA or ANCOVA.

## Supplementary Materials

The following are available online at https://www.mdpi.com/2072-6643/12/6/1831/s1. Table S1: CONSORT 2010 checklist of information to include when reporting a randomized trial.
